# Thoracic Segmental Spinal Anesthesia for Emergency Cholecystectomy: A Case Report

**DOI:** 10.7759/cureus.30184

**Published:** 2022-10-11

**Authors:** Yahya M Aljuba, Adham M Amro, Amro T Alkadi, Husam Taamrah, Majde G Hamamdh

**Affiliations:** 1 Anesthesia and Critical Care, Al-Ahli Hospital, Hebron, PSE

**Keywords:** case report, surgery, cholecystectomy, copd, spinal, regional anesthesia

## Abstract

Anesthesia is a rapidly evolving medical specialty, centered around the patient’s safety, and enhancement of surgical outcomes. In their daily practice, anesthesiologists encounter complex challenges, and always search for, and practice the best evidence-based medicine, which allows them to overcome the challenges, and to obtain the maximum benefits of the intervention, with minimal side effects. Regional anesthesia is considered a favorable modality with significant potential in selected patients and is becoming more popular day by day. In this regard, we present a case report of a 65-year-old man, with multiple co-morbidities, who needed urgent surgery to remove a gangrenous gall bladder. After discussion with the surgical colleagues, thoracic segmental spinal anesthesia (SA) was performed, and the patient underwent an open cholecystectomy awake with spontaneous respiration. The procedure went well, and the patient recovered quickly. This case presentation highlights the potential benefits of segmental SA in managing some selected cases.

## Introduction

For upper abdominal or thoracic surgeries, patients who have a preoperative pulmonary impairment, such as those with severe chronic obstructive pulmonary disease (COPD), are significantly more susceptible to postoperative respiratory failure than otherwise healthy patients [1]. General anesthesia (GA) has a higher risk of complications even in the general population, and more so in those with co-morbidities. Perioperative ventilation and oxygenation may be adversely affected by atelectasis and ventilation-perfusion mismatch, and also may be augmented by the influences of residual anesthesia, immobilization, and administration of analgesic and sedative drugs [2,3]. This can result in a number of intraoperative and postoperative complications such as bronchospasm, laryngospasm, and prolonged mechanical ventilation. Proper choice of anesthetic technique in these patients is necessary to reduce the risk of these complications [4]. One of the anesthetic techniques that may be used for such patients in procedures such as cholecystectomy is thoracic spinal anesthesia (SA) [5]. We report a case of a severe COPD patient (GOLD 4 class) who had a successful emergency cholecystectomy under segmental thoracic SA.

## Case presentation

In this case, we present a 65-year-old man, ASA (American Society of Anesthesiologists) physical class III, who used to be a truck driver, but left his job due to his medical condition, ex-smoker for four years (112 pack/year), morbidly obese (body mass index: 42 kg/m^2^, obesity class III), known case of COPD for 13 years, on domiciliary oxygen therapy via nasal cannula 2 liter/minute (L/M) eight hours per day. His surgical history was significant for an open appendectomy at age of 18 and an umbilical hernia repair at age of 47, both were performed uneventfully under GA. His drug history included inhaled corticosteroids, long-acting beta-agonists, and an oral mucolytic. The patient had limited exercise tolerance due to his obesity and co-morbidities, he developed shortness of breath on low-intensity exercises, for example, he was able to eat, brush his hair, and dress, but became short of breath after walking 20-30 meters on the floor level.

The patient's complaint started one week before admission when he developed acute right upper quadrant (RUQ) abdominal pain. So, he sought medical advice at a local small-town hospital. Initial assessment at the emergency department showed that the patient was conscious, oriented, and connected to oxygen via nasal cannula. His vital signs were as follows: Blood Pressure (BP): 119/76 mmHg, heart rate (HR): 107 beats per minute (PBM), O_2_ saturation: 91% on nasal cannula 3 L/M, temperature: 37.4 degrees Celsius and respiratory rate of 21 cycle per Minute (C/M). Chest auscultation revealed decreased air entry bilaterally, with inspiratory and expiratory wheezes. Abdomen examination revealed RUQ tenderness with positive Rovsing’s sign, as well as healed scars of an old umbilical hernia repair and appendectomy surgeries. Further workup was done; blood tests showed leukocytosis, eosinophilia, and high C-reactive protein level. Abdomen ultrasound showed a 2 cm gallbladder stone impacted in the neck of the gallbladder, with peri-cystic edema and wall thickening.

The patient was admitted to the hospital overnight and given intravenous fluids and antibiotics. The surgeon’s decision was to proceed with cholecystectomy, however; due to the patient's co-morbidities, and after consulting the anesthesia team; a decision was made to transfer the patient to a tertiary center with surgical intensive care and pulmonary units, so the patient was transferred to our hospital via an ambulance and with a medical companion, the patient arrived in our hospital after two hours road trip.

Clinical examination and diagnostic tests

**Figure 1:**
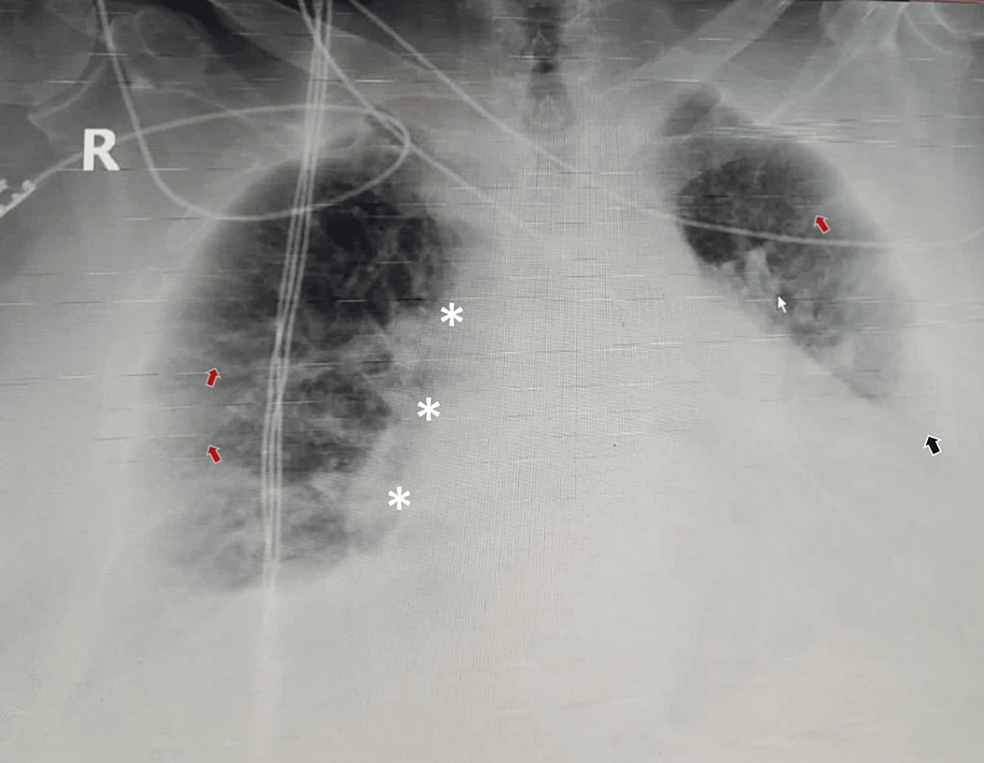
Chest x-ray of the patient shows bilateral infiltrations with diffuse linear increased interstitial markings throughout both lungs (red arrows), shaggy outline around the heart (*), and obliteration of left costo-phrenic angle (black arrow).

**Figure 2:**
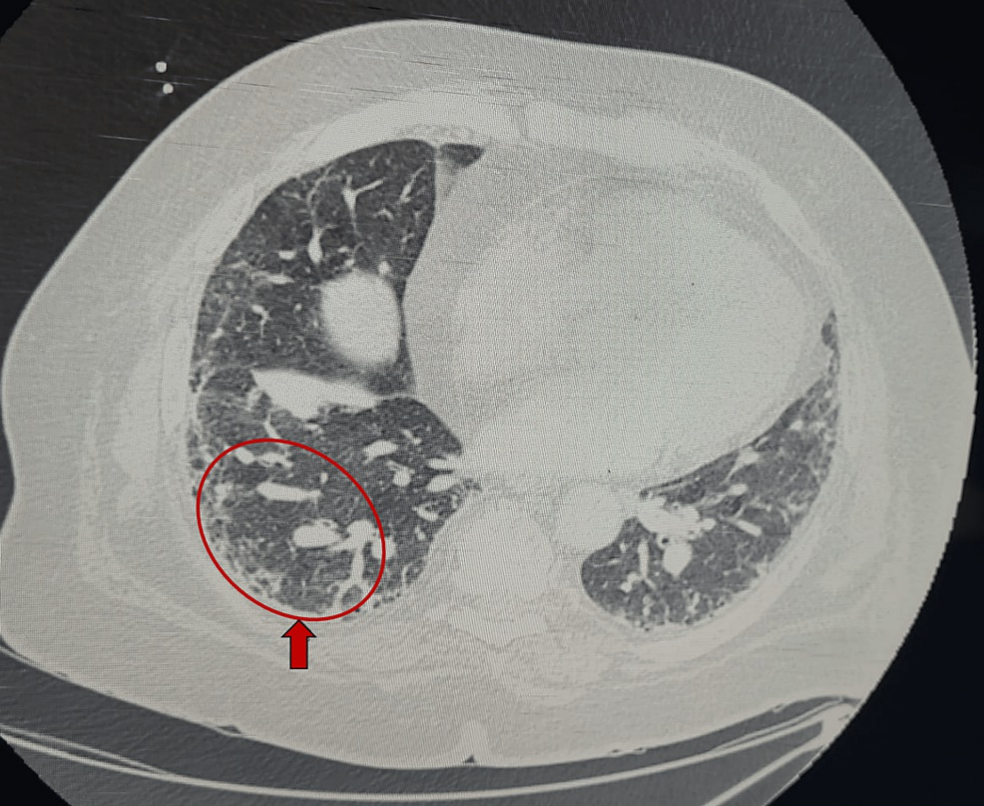
Chest CT scan of the patient showing peripheral honeycombing (red arrow and circle) consistent with pulmonary fibrosis.

ِِAfter admission to the surgical ICU, the patient was evaluated by a multidisciplinary team, including a pulmonologist, intensivist, anesthesiologist and surgeon. The patient was conscious and oriented, moderately dyspneic and tachypneic at rest (modified Medical Research Council [mMRC] dyspnea scale grade 3) on O_^2^_ via nasal cannula 2 L/M. He was able to tell a sentence of 4-5 words before breathing again. He was not cyanosed. His vital signs on admission were as follows: BP: 107/63 mmHg, HR: 91 PBM, O2 saturation: 93% on nasal cannula 3 L/M, temperature: 37.1 degree Celsius and respiratory rate of 19 C/M. His chest auscultation revealed bilateral dry crepitation, with inspiratory and expiratory wheezes more on the left side. Chest x-ray (Figure 1) showed bilateral infiltrations with diffuse increased interstitial markings throughout both lungs, shaggy outline around the heart and blunting of left costo-phrenic angle consistent with pleural effusion or left lower lobe collapse. Chest and abdomen computerized tomography (CT) scan showed early pulmonary fibrosis (Figure 2) with the dilated pulmonary trunk, perforated GB with stone at the ampulla of Vater. Transthoracic echocardiography showed preserved systolic and diastolic functions with pulmonary hypertension (ejection fraction 60% and pulmonary artery pressure of 55 mmHg). His electrocardiogram (ECG) showed regular sinus rhythm with frequent premature atrial contractions, and baseline arterial blood gases (ABGs) on O_2_ therapy 2 L/M were suggestive of hypoxic, hypercapnic compensated respiratory acidosis (Table 1).

**Table 1:** Patient's blood gases report.

Variable	Result
pH	7.38
paCO2	60.8 mmHg
paO2	57 mmHg
HCO3	38 mmol/L
O2 sat	88.5%
lactate	0.4 mmol/L

The pulmonologist evaluated the patient thoroughly and tried to optimize his condition with inhaled bronchodilators and steroids, mucolytics, and incentive spirometry; however, his opinion was that the patient’s condition is not fully reversible and could only be partially improved.

After a multidisciplinary team discussion, the decision was made to perform combined spinal-epidural anesthesia and open cholecystectomy. We decided to do segmental SA with target sensory loss from T6 to T10, to decrease the complications (such as hypotension, urine retention, or high SA level), and speed the recovery. A rescue GA plan was ready to be performed if needed. However, the patient had concerns regarding the epidural analgesia and preferred to undergo the least possible invasive intervention. Consequently, the patient gave us consent to perform SA without approving epidural analgesia. Open cholecystectomy was opted rather than laparoscopic approach due to extensive tissue inflammation and necrosis, with perforation of gall bladder, in addition to the undesirable effects of pneumoperitoneum on the patient's respiratory and cardiovascular systems.

After discussing the case with the patient and his family, and obtaining informed written consent form, we transferred the patient to the operation room. Standard monitoring was applied (non-invasive BP, ECG, pulse oximetry, temperature), two intravenous cannulas inserted (18 and 20 gauge) and right radial arterial catheter inserted under sterile technique for invasive BP monitoring. Baseline values of vital signs recorded. Then, under complete aseptic conditions, in sitting position, lidocaine 1% 2 mL was used for local skin infiltration, then at T8-T9 level, a pencil point spinal needle 27G with an introducer (B. Braun Pencan ^TM^, Melsungen, Germany) introduced using midline approach until free drainage of CSF obtained. isobaric bupivacaine (Grindex ^TM^, Bupivacaine-Grindeks 5 mg/mL, Riga, Latvia) 7.5 mg (volume of 1.5 mL), with dexmedetomidine 5 mcg (Precedex ^TM^, Pfizer, New York, USA) diluted in a volume of 0.5 mL were injected gently without complications (total injectate volume of 2 mL). Isobaric bupivacaine was used due to expert’s opinion regarding segmental SA. Our anesthesia consultant advised to use isobaric bupivacaine because the sensory loss level target was for specific dermatomes, while hyperbaric bupivacaine is preferred for lower abdominal procedures. Intrathecal dexmedetomidine was added to prolong the duration and improve the quality of SA. After drugs injection, the patient kept in sitting position for one minute, to ensure isobaric bupivacaine solidification at the same level of injection, then the patient was positioned in 30 degrees head up position due to his obesity and respiratory issues. The sensation was assessed with pin-prick test every one minute, and a sensory block between the levels of T6-T10 was confirmed after three minutes of intrathecal local anesthetic injection. Motor functions of lower limbs were not affected significantly (lower limbs muscle power 3/5), this was attributed to the low dose of local anesthetic injected. Patient kept on O_2_ therapy via nasal cannula 3 L/M. No supplementary sedation was given. Verbal contact with the patient continued throughout the procedure, the patient was reassured and encouraged to report any complaints instantly. To make clear cutpoints for intervention; standard definitions of vital signs were agreed to be as follows: hypotension was defined as 20% drop of mean arterial pressure (MAP) below the baseline, and bradycardia was defined as HR below 60 PBM.

Surgery course and outcome

Clinical observation and careful hemodynamic monitoring were both used to continuously monitor the patient throughout the procedure (ECG, HR, BP, pulse oximetry, respiratory rate and ABGs). All data were recorded at five-minute intervals, with exception of blood gases which were obtained every 15 minutes. Blood pressure decreased from 155/89 mmHg to the lowest value of 87/59 mmHg without bradycardia, nausea, vomiting, or chest discomfort. Hypotension responded well to intravenous boluses of phenylephrine (total 150 mcg) and intravenous fluids; blood pressure raised to 110/70 mmHg and stabilized. Apart from intravenous fluids and phenylephrine, no further medications were administered. The dermatome level of anesthesia was established from T6 to T10, partially preserving the movement and sensation of the lower limbs. After ensuring adequate sensory anesthesia via pin-prick test, sterilization and draping were done, a Kocher incision was made, surgical team found a gangrenous perforated gall bladder, calculus cholecystitis and very friable tissue, open cholecystectomy was performed, and a drain was inserted to drain any fluid or pus collections. The surgery lasted 50 minutes and was uneventful despite few episodes of cough (which were expected due to the patient's position and respiratory disease). Post-operatively, the patient was transferred to the surgical ICU and kept under close observation. Sensory test was done regularly every 15 minutes, and the patient started to restore sensation after about 130 minutes after introduction of spinal anesthetic, he was hemodynamically stable. After the resolution of the block's effect, the patient's pain was controlled with regular IV paracetamol every 6 hours, with additional supplementation of ketorolac every 8 hours if needed. Opioids were considered the last resort and were not needed. On the next day, the patient was transferred to the surgical ward and started on an oral diet. 

Discharge and follow up

The patient was followed closely during his hospitalization; his post-operative course was smooth. On post-operative day 7, the patient was evaluated by his surgeon and pulmonologist, the patient was found to be in stable condition, with stable vital signs. His O2 saturation was 90-92% on O_2_ therapy via nasal cannula 2 L/M (his baseline saturation), Richmond Agitation-Sedation Scale (RASS) score of zero, Numerical Rating Scale (NRS) pain score of less than 3. Mild nausea (grade 2) decreased inflammatory markers, and no surgical events such as bleeding or wound dehiscence. So, the patient was considered to be fit for discharge. As the patient was referred from a faraway hospital; he was given a medical report describing his medical condition, with instructions for follow-up at his town's hospital. Direct contact was made with the referring hospital via phone call to arrange the patient's follow-up. Additionally, the patient was given a phone number to contact when needed. The abdomen drain was removed, and the patient was discharged home.

## Discussion

Abdominal surgeries are often risky and may be associated with post-operative pulmonary complications (e.g., atelectasis, pneumonia, ICU admission, need for mechanical ventilation, and prolonged hospitalization). These complications increase peri-operative mortality and morbidity, especially in patients with respiratory diseases. Therefore, a careful pre-anesthesia evaluation should be performed on all patients undergoing abdominal surgery, especially those who have chronic respiratory diseases [6].

The prevention of postoperative pulmonary complications or worsening of the underlying disorders is the primary focus for anesthetic care in patients with respiratory issues. Pre-anesthesia respiratory function testing and the patient's general condition optimization are essential. Additionally, the best anesthetic approach must be chosen to mitigate the anesthesia effects on respiratory function [7].

There are two modes of anesthesia during cholecystectomy procedures: regional (spinal or epidural) and general. GA is the most popular and sensible option as it has relative benefits over regional anesthesia, especially the need for sufficient muscle relaxation for the surgery. However, in individuals with coexisting cardiopulmonary problems, SA is recommended to the GA [8]. During major abdominal surgeries, regional anesthesia considerably lowers the incidence of pulmonary complications in COPD patients [9].

Tracheal intubation, mechanical ventilation, and the use of neuromuscular blockers are all necessary for GA. These techniques can cause a number of complications including bronchospasm, ventilation-perfusion mismatch, lung infiltration, atelectasis, and decreased respiratory muscle function due to residual muscle relaxation. Positive pressure ventilation also impairs the venous return to the heart and reduces cardiac output. Regional anesthesia is sufficient for abdominal surgeries and minimizes the risk of these problems [10].

Thoracic SA has many complications, such as spinal cord trauma, infection (e.g., meningitis), cardiovascular depression, high SA, urine retention, and respiratory compromise.

In our case, the patient had severely compromised pulmonary functions due to a pre-existing COPD. Given the patient's pulmonary condition and the estimated surgery duration of one hour, segmental thoracic SA was performed with good results.

After reviewing the literature about similar cases and contacting authors of case reports for patients similar to our patient's case, the decision to perform thoracic segmental SA was made for the benefits mentioned above. Specific benefits for that choice in our case included superior pain control to opioids, and avoidance of opioid side-effects after the procedure (e.g., respiratory depression, urine retention, nausea and vomiting, and delayed bowel function recovery). Furthermore, the decreased hospitalization period offers an economic advantage for the chosen segmental SA over the GA [11,12].

## Conclusions

Although thoracic segmental SA is not used routinely and has some limitations when used for abdominal surgeries (e.g., hypotension, risk of spinal cord injury, high SA level, and patient's discomfort); this case report demonstrates that segmental thoracic SA is a reasonable substitute for the use of conventional GA during open cholecystectomy in patients with pre-existing respiratory compromise and major abdominal surgery. It is safe and more effective than GA in providing pain-free intervals after surgery, reducing the need for analgesics and opioids, and lowering the risk of postoperative pulmonary complications. Although each patient is unique, and there is a wide set of anesthetic options when managing conventional cases; the scenario described in this case report can help anesthesiologists in expanding their boundaries when managing complex cases.
